# Supplementary material for: Characterization and Effect of Refining on the Oil Extracted from Durum Wheat By-Products
Source: Foods. 2022 Feb 25;11(5):683. doi: 10.3390/foods11050683 (PMC8909574; doi:10.3390/foods11050683)
Supplement: Supplementary file 1 [file foods-11-00683-s001.zip › foods-1583546-supplementary.pdf]

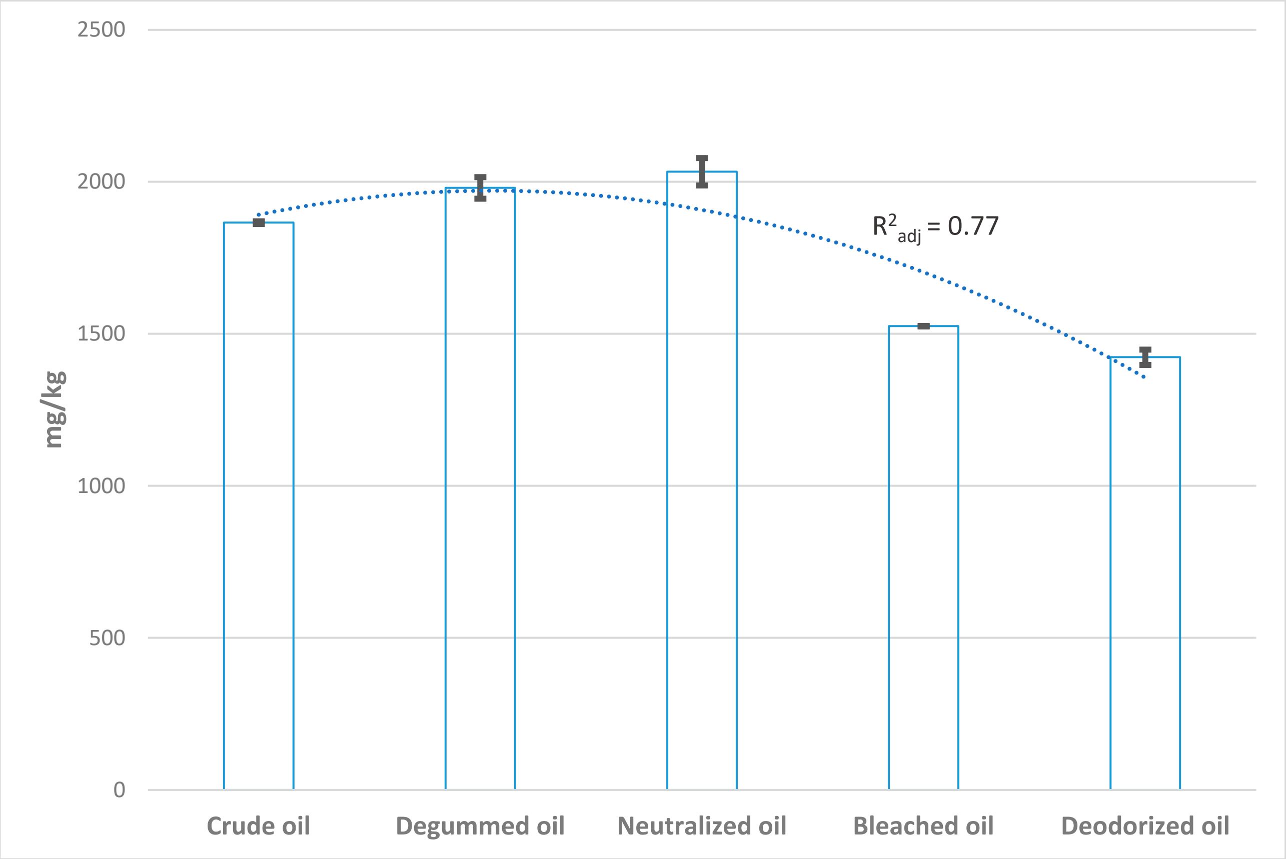

Figure S1. Second order polynomial fitting to the evolution of total vitamin E content during refining. The error bars represent the standard deviation (n = 2).
